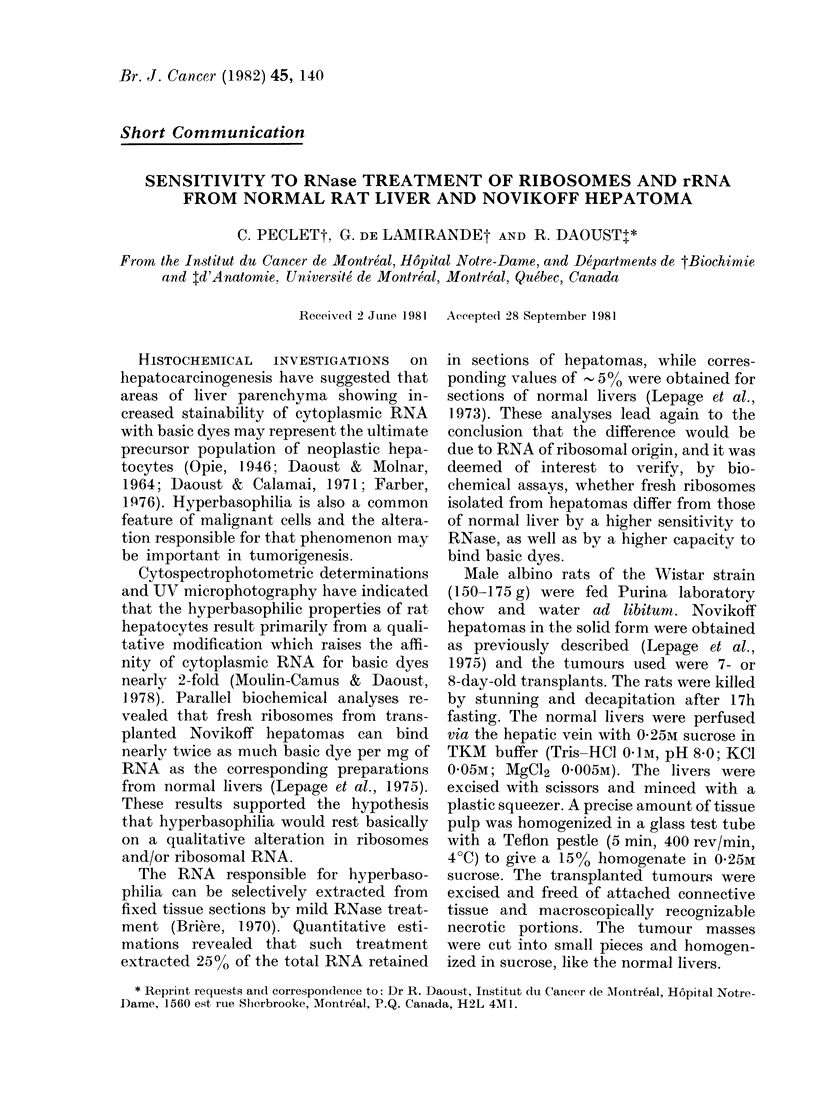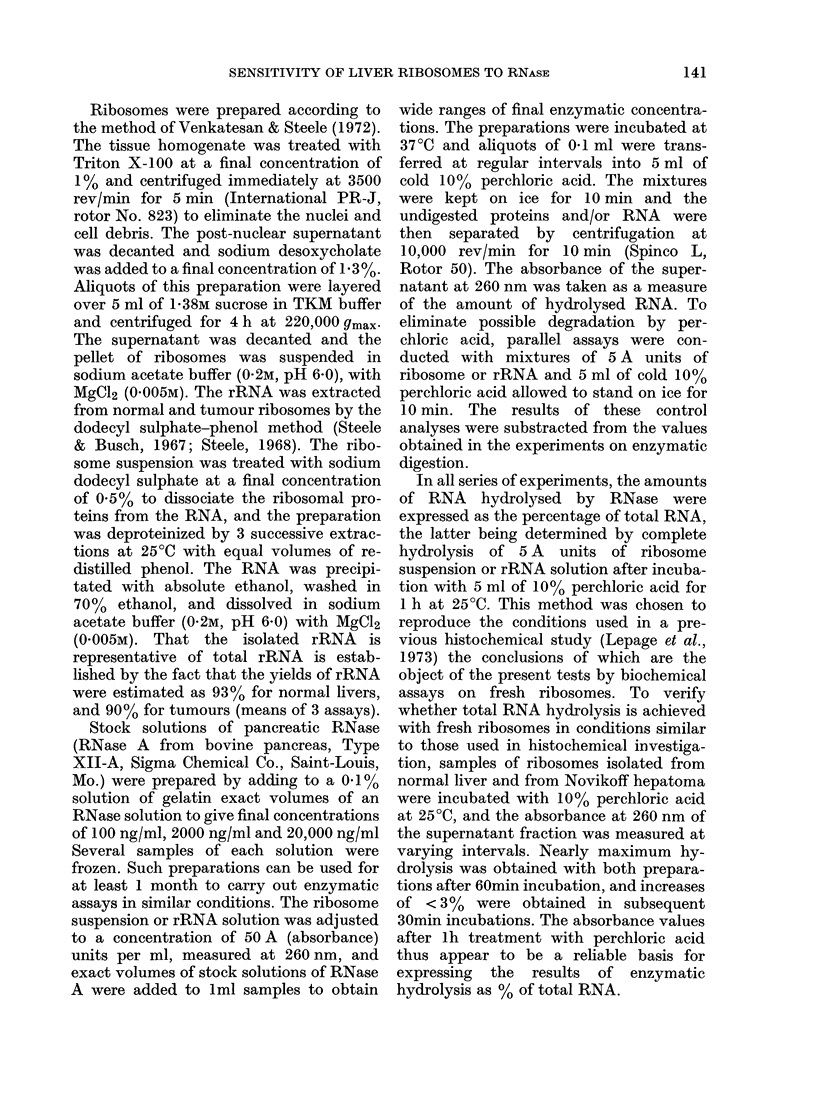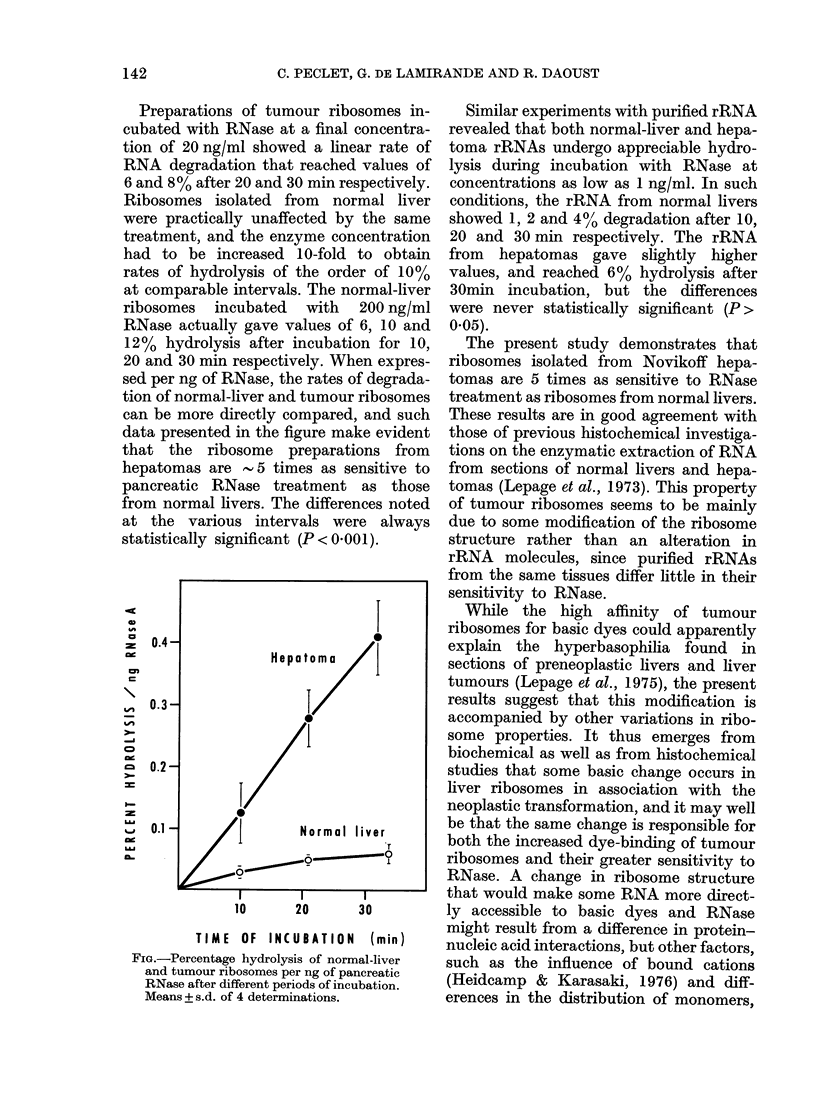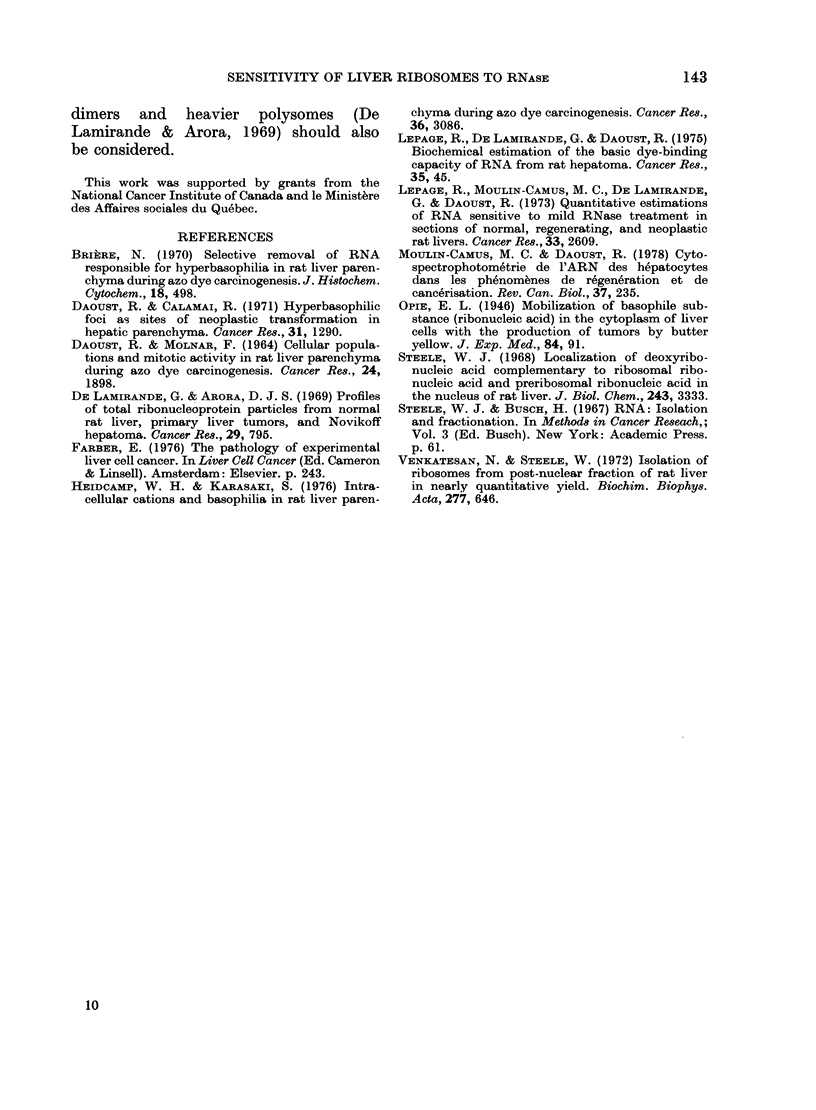# Sensitivity to RNase treatment of ribosomes and rRNA from normal rat liver and Novikoff hepatoma.

**DOI:** 10.1038/bjc.1982.17

**Published:** 1982-01

**Authors:** C. Peclet, G. de Lamirande, R. Daoust


*Br. J. Cancer* (1982) **45**, 140

***Short Communication***

### SENSITIVITY TO RNase TREATMENT OF RIBOSOMES AND rRNA FROM NORMAL RAT LIVER AND NOVIKOFF HEPATOMA

C. PECLET†, G. DE LAMIRANDE† AND R. DAOUST‡*

*From the Institut du Cancer de Montréal, Hôpital Notre-Dame, and Départments de †Biochimie and ‡d'Anatomie, Université de Montréal, Montréal, Québec, Canada*

Received 2 June 1981 Accepted 28 September 1981

HISTOCHEMICAL INVESTIGATIONS on hepatocarcinogenesis have suggested that areas of liver parenchyma showing increased stainability of cytoplasmic RNA with basic dyes may represent the ultimate precursor population of neoplastic hepatocytes (Opie, 1946; Daoust & Molnar, 1964; Daoust & Calamai, 1971; Farber, 1976). Hyperbasophilia is also a common feature of malignant cells and the alteration responsible for that phenomenon may be important in tumorigenesis.

Cytospectrophotometric determinations and UV microphotography have indicated that the hyperbasophilic properties of rat hepatocytes result primarily from a qualitative modification which raises the affinity of cytoplasmic RNA for basic dyes nearly 2-fold (Moulin-Camus & Daoust, 1978). Parallel biochemical analyses revealed that fresh ribosomes from transplanted Novikoff hepatomas can bind nearly twice as much basic dye per mg of RNA as the corresponding preparations from normal livers (Lepage *et al.*, 1975). These results supported the hypothesis that hyperbasophilia would rest basically on a qualitative alteration in ribosomes and/or ribosomal RNA.

The RNA responsible for hyperbasophilia can be selectively extracted from fixed tissue sections by mild RNase treatment (Brière, 1970). Quantitative estimations revealed that such treatment extracted 25% of the total RNA retained in sections of hepatomas, while corresponding values of ~5% were obtained for sections of normal livers (Lepage *et al.*, 1973). These analyses lead again to the conclusion that the difference would be due to RNA of ribosomal origin, and it was deemed of interest to verify, by biochemical assays, whether fresh ribosomes isolated from hepatomas differ from those of normal liver by a higher sensitivity to RNase, as well as by a higher capacity to bind basic dyes.

Male albino rats of the Wistar strain (150–175 g) were fed Purina laboratory chow and water *ad libitum*. Novikoff hepatomas in the solid form were obtained as previously described (Lepage *et al.*, 1975) and the tumours used were 7- or 8-day-old transplants. The rats were killed by stunning and decapitation after 17h fasting. The normal livers were perfused *via* the hepatic vein with 0·25M sucrose in TKM buffer (Tris–HCl 0·1M, pH 8·0; KCl 0·05M; $MgCl_2$ 0·005M). The livers were excised with scissors and minced with a plastic squeezer. A precise amount of tissue pulp was homogenized in a glass test tube with a Teflon pestle (5 min, 400 rev/min, 4°C) to give a 15% homogenate in 0·25M sucrose. The transplanted tumours were excised and freed of attached connective tissue and macroscopically recognizable necrotic portions. The tumour masses were cut into small pieces and homogenized in sucrose, like the normal livers.

* Reprint requests and correspondence to: Dr R. Daoust, Institut du Cancer de Montréal, Hôpital Notre-Dame, 1560 est rue Sherbrooke, Montréal, P.Q. Canada, H2L 4M1.

Ribosomes were prepared according to the method of Venkatesan & Steele (1972). The tissue homogenate was treated with Triton X-100 at a final concentration of 1% and centrifuged immediately at 3500 rev/min for 5 min (International PR-J, rotor No. 823) to eliminate the nuclei and cell debris. The post-nuclear supernatant was decanted and sodium desoxycholate was added to a final concentration of 1·3%. Aliquots of this preparation were layered over 5 ml of 1·38M sucrose in TKM buffer and centrifuged for 4 h at 220,000 $g_{max}$. The supernatant was decanted and the pellet of ribosomes was suspended in sodium acetate buffer (0·2M, pH 6·0), with $MgCl_2$ (0·005M). The rRNA was extracted from normal and tumour ribosomes by the dodecyl sulphate–phenol method (Steele & Busch, 1967; Steele, 1968). The ribosome suspension was treated with sodium dodecyl sulphate at a final concentration of 0·5% to dissociate the ribosomal proteins from the RNA, and the preparation was deproteinized by 3 successive extractions at 25°C with equal volumes of redistilled phenol. The RNA was precipitated with absolute ethanol, washed in 70% ethanol, and dissolved in sodium acetate buffer (0·2M, pH 6·0) with $MgCl_2$ (0·005M). That the isolated rRNA is representative of total rRNA is established by the fact that the yields of rRNA were estimated as 93% for normal livers, and 90% for tumours (means of 3 assays).

Stock solutions of pancreatic RNase (RNase A from bovine pancreas, Type XII-A, Sigma Chemical Co., Saint-Louis, Mo.) were prepared by adding to a 0·1% solution of gelatin exact volumes of an RNase solution to give final concentrations of 100 ng/ml, 2000 ng/ml and 20,000 ng/ml Several samples of each solution were frozen. Such preparations can be used for at least 1 month to carry out enzymatic assays in similar conditions. The ribosome suspension or rRNA solution was adjusted to a concentration of 50 A (absorbance) units per ml, measured at 260 nm, and exact volumes of stock solutions of RNase A were added to 1ml samples to obtain wide ranges of final enzymatic concentrations. The preparations were incubated at 37°C and aliquots of 0·1 ml were transferred at regular intervals into 5 ml of cold 10% perchloric acid. The mixtures were kept on ice for 10 min and the undigested proteins and/or RNA were then separated by centrifugation at 10,000 rev/min for 10 min (Spinco L, Rotor 50). The absorbance of the supernatant at 260 nm was taken as a measure of the amount of hydrolysed RNA. To eliminate possible degradation by perchloric acid, parallel assays were conducted with mixtures of 5 A units of ribosome or rRNA and 5 ml of cold 10% perchloric acid allowed to stand on ice for 10 min. The results of these control analyses were substracted from the values obtained in the experiments on enzymatic digestion.

In all series of experiments, the amounts of RNA hydrolysed by RNase were expressed as the percentage of total RNA, the latter being determined by complete hydrolysis of 5 A units of ribosome suspension or rRNA solution after incubation with 5 ml of 10% perchloric acid for 1 h at 25°C. This method was chosen to reproduce the conditions used in a previous histochemical study (Lepage *et al.*, 1973) the conclusions of which are the object of the present tests by biochemical assays on fresh ribosomes. To verify whether total RNA hydrolysis is achieved with fresh ribosomes in conditions similar to those used in histochemical investigation, samples of ribosomes isolated from normal liver and from Novikoff hepatoma were incubated with 10% perchloric acid at 25°C, and the absorbance at 260 nm of the supernatant fraction was measured at varying intervals. Nearly maximum hydrolysis was obtained with both preparations after 60min incubation, and increases of $< 3\%$ were obtained in subsequent 30min incubations. The absorbance values after 1h treatment with perchloric acid thus appear to be a reliable basis for expressing the results of enzymatic hydrolysis as % of total RNA.

Preparations of tumour ribosomes incubated with RNase at a final concentration of 20 ng/ml showed a linear rate of RNA degradation that reached values of 6 and 8% after 20 and 30 min respectively. Ribosomes isolated from normal liver were practically unaffected by the same treatment, and the enzyme concentration had to be increased 10-fold to obtain rates of hydrolysis of the order of 10% at comparable intervals. The normal-liver ribosomes incubated with 200 ng/ml RNase actually gave values of 6, 10 and 12% hydrolysis after incubation for 10, 20 and 30 min respectively. When expressed per ng of RNase, the rates of degradation of normal-liver and tumour ribosomes can be more directly compared, and such data presented in the figure make evident that the ribosome preparations from hepatomas are ~5 times as sensitive to pancreatic RNase treatment as those from normal livers. The differences noted at the various intervals were always statistically significant ($P < 0{\cdot}001$).

Fig.—Percentage hydrolysis of normal-liver and tumour ribosomes per ng of pancreatic RNase after different periods of incubation. Means ± s.d. of 4 determinations.

Similar experiments with purified rRNA revealed that both normal-liver and hepatoma rRNAs undergo appreciable hydrolysis during incubation with RNase at concentrations as low as 1 ng/ml. In such conditions, the rRNA from normal livers showed 1, 2 and 4% degradation after 10, 20 and 30 min respectively. The rRNA from hepatomas gave slightly higher values, and reached 6% hydrolysis after 30min incubation, but the differences were never statistically significant ($P > 0{\cdot}05$).

The present study demonstrates that ribosomes isolated from Novikoff hepatomas are 5 times as sensitive to RNase treatment as ribosomes from normal livers. These results are in good agreement with those of previous histochemical investigations on the enzymatic extraction of RNA from sections of normal livers and hepatomas (Lepage *et al.*, 1973). This property of tumour ribosomes seems to be mainly due to some modification of the ribosome structure rather than an alteration in rRNA molecules, since purified rRNAs from the same tissues differ little in their sensitivity to RNase.

While the high affinity of tumour ribosomes for basic dyes could apparently explain the hyperbasophilia found in sections of preneoplastic livers and liver tumours (Lepage *et al.*, 1975), the present results suggest that this modification is accompanied by other variations in ribosome properties. It thus emerges from biochemical as well as from histochemical studies that some basic change occurs in liver ribosomes in association with the neoplastic transformation, and it may well be that the same change is responsible for both the increased dye-binding of tumour ribosomes and their greater sensitivity to RNase. A change in ribosome structure that would make some RNA more directly accessible to basic dyes and RNase might result from a difference in protein–nucleic acid interactions, but other factors, such as the influence of bound cations (Heidcamp & Karasaki, 1976) and differences in the distribution of monomers, dimers and heavier polysomes (De Lamirande & Arora, 1969) should also be considered.

This work was supported by grants from the National Cancer Institute of Canada and le Ministère des Affaires sociales du Québec.

### REFERENCES

Brière, N. (1970) Selective removal of RNA responsible for hyperbasophilia in rat liver parenchyma during azo dye carcinogenesis. *J. Histochem. Cytochem.*, **18**, 498.

Daoust, R. & Calamai, R. (1971) Hyperbasophilic foci as sites of neoplastic transformation in hepatic parenchyma. *Cancer Res.*, **31**, 1290.

Daoust, R. & Molnar, F. (1964) Cellular populations and mitotic activity in rat liver parenchyma during azo dye carcinogenesis. *Cancer Res.*, **24**, 1898.

De Lamirande, G. & Arora, D. J. S. (1969) Profiles of total ribonucleoprotein particles from normal rat liver, primary liver tumors, and Novikoff hepatoma. *Cancer Res.*, **29**, 795.

Farber, E. (1976) The pathology of experimental liver cell cancer. In *Liver Cell Cancer* (Ed. Cameron & Linsell). Amsterdam: Elsevier. p. 243.

Heidcamp, W. H. & Karasaki, S. (1976) Intracellular cations and basophilia in rat liver parenchyma during azo dye carcinogenesis. *Cancer Res.*, **36**, 3086.

Lepage, R., De Lamirande, G. & Daoust, R. (1975) Biochemical estimation of the basic dye-binding capacity of RNA from rat hepatoma. *Cancer Res.*, **35**, 45.

Lepage, R., Moulin-Camus, M. C., De Lamirande, G. & Daoust, R. (1973) Quantitative estimations of RNA sensitive to mild RNase treatment in sections of normal, regenerating, and neoplastic rat livers. *Cancer Res.*, **33**, 2609.

Moulin-Camus, M. C. & Daoust, R. (1978) Cytospectrophotométrie de l'ARN des hépatocytes dans les phénomènes de régenération et de cancérisation. *Rev. Can. Biol.*, **37**, 235.

Opie, E. L. (1946) Mobilization of basophile substance (ribonucleic acid) in the cytoplasm of liver cells with the production of tumors by butter yellow. *J. Exp. Med.*, **84**, 91.

Steele, W. J. (1968) Localization of deoxyribonucleic acid complementary to ribosomal ribonucleic acid and preribosomal ribonucleic acid in the nucleus of rat liver. *J. Biol. Chem.*, **243**, 3333.

Steele, W. J. & Busch, H. (1967) RNA: Isolation and fractionation. In *Methods in Cancer Reseach*,; Vol. 3 (Ed. Busch). New York: Academic Press. p. 61.

Venkatesan, N. & Steele, W. (1972) Isolation of ribosomes from post-nuclear fraction of rat liver in nearly quantitative yield. *Biochim. Biophys. Acta*, **277**, 646.